# Quality and readability of web-based information on dental caries in Arabic: an infodemiological study

**DOI:** 10.1186/s12903-023-03547-1

**Published:** 2023-10-25

**Authors:** Khalid Aboalshamat

**Affiliations:** Dental Public Health Division, Preventative Dentistry Department, College of Dentistry, Umm Al-Qura University, Makkah, Saudi Arabia

**Keywords:** Quality, Readability, Web-based information, Dental caries, Arabic, Infodemiological study

## Abstract

**Background:**

Web-based information on dental caries in Arabic remains poorly understood. This study aimed to assess the quality and readability of web-based information about dental caries in Arabic.

**Methods:**

The first 100 websites in Arabic about dental caries were retrieved from Google and Bing using common terms. The websites were classified and evaluated for quality based on the Journal of the American Medical Association (JAMA) benchmark criteria, the DISCERN tool, and the presence of the Health on the Net Foundation Code of Conduct (HONcode). Readability was assessed using online readability indexes.

**Results:**

A total of 102 Arabic websites were included. The JAMA benchmark score was low (m = 0.36, SD = 0.56), with 67.7% failing to meet any of the JAMA criteria. The DISCERN total score mean was 37.68 (SD = 7.99), with a majority (67.65%) of moderate quality. None of the websites had the HONcode. Readability was generally good, with 52.94% of websites having a Flesch–Kincaid Grade Level (FKGL) < 7, 91.18% having a Simple Measure of Gobbledygook (SMOG) < 7, and 85.29% having a Flesch reading ease (FRE) score ≥ 80. There was a positive correlation between JAMA and DISCERN scores (*p* < 0.001). DISCERN scores were positively correlated with the number of words (*p* < 0.001) and sentences (*p* = 0.004) on the websites. However, JAMA or DISCERN scores were not correlated with FKGL, SMOG, or FRE scores (*p* > 0.05).

**Conclusions:**

The quality of Arabic dental caries websites was found to be low, despite their readability. Efforts are needed to introduce more reliable sources for discussing dental caries and treatment options on sites aimed at Arabic populations.

**Supplementary Information:**

The online version contains supplementary material available at 10.1186/s12903-023-03547-1.

## Background

According to the World Health Organization [1], 2 billion adults and 514 million children have permanent and primary tooth caries, which makes dental caries the most prevalent health condition worldwide [2]. Dental caries is defined by the American Dental Association (ADA) as a complex and dynamic disease that involves biofilm formation, sugar consumption, and multiple contributing factors. Caries is characterized by the cyclic process of demineralization and remineralization of dental hard tissues [3]. The total economic burden of untreated caries in permanent teeth amounted to $21.19 billion and for deciduous teeth, $0.90 billion, which represented 11% and 0.5%, respectively, of the overall cost of dental disease worldwide [4]. A systematic review indicated that dental caries is negatively related to the quality of life [5], so it was recommended that it is of the utmost importance to enhance comprehension of the mechanisms involved, with a particular emphasis on preventive measures and appropriate therapeutic interventions to help reduce this global burden [6].

Today, a search of the internet might be one of the main sources of medical information worldwide [7–9]. In fact, many patients (45–85%) bring information they have searched for online to medical visits [10], while 28.2% search for medical information because they do not trust their physicians, according to a national representative French study [11]. This may be accentuated by the COVID-19 pandemic, which caused people to search the internet for answers to their questions about the disease [12–14]. However, the spread of misinformation on the internet is a major concern that has been reported to be a global phenomenon [15, 16]. It is a serious problem that can affect people’s quality of life and may lead to increased mortality rates [16], which underscores the need to enforce legislation, increase public awareness, and improve available health-related information [15].

Many studies have assessed the quality and readability of English-language websites providing information on oral diseases and conditions such as burning mouth syndrome [17], dental implants [18, 19], treatment of the mouth in systemic sclerosis [20], oral leukoplakia [21], oral lichen planus [22], and many others [23]. Similar studies have been conducted in other languages, such as Portuguese [24], Spanish [25], Danish [26], French [27] and others [23].

The limited number of studies in Arabic regarding oral conditions have investigated only periodontal disease [28], oral cancer [29], denture hygiene [30], and dental implants [31]. These studies were assessed using mainly three items; (1) the presence of the Health on the Net (HON) Foundation Code of Conduct (HONcode) [32], which indicates that a website is following HON criteria, (2) the DISCERN tool that measures the quality of a website [33], and (3) readability of the website using readability calculator tools. The studies [28–31] found only a few websites (1.5–7.1%) displaying the HONcode and 2.2% to 4.6% with a high DISCERN score. However, the websites were mostly simple and readable.

Yet, web-based information in Arabic regarding other oral conditions and diseases, such as dental caries, is lacking. The Arabic language is the fifth most commonly spoken language, with more than 422 million people speaking Arabic and 22 countries with Arabic as their official language [34]. Assessing web-based information regarding oral conditions and disease is important for evaluating the current status of the public’s highly accessible sources of information. Thus, this study aimed to assess the quality and readability of web‑based Arabic information about dental caries.

## Methods

### Search strategy

This was an infodemiological study using two search engines. Google Chrome version 114.0.5735.110 (http://www.google.com), the most frequently used search engine, was used in incognito mode to minimize the influence of search histories and personalized search algorithms on the results [35]. Also used was Bing (http://www.bing.com), Microsoft’s search engine that has recently incorporated a chatbot [36]. The search was conducted on February 26, 2023.

**Fig. 1:**
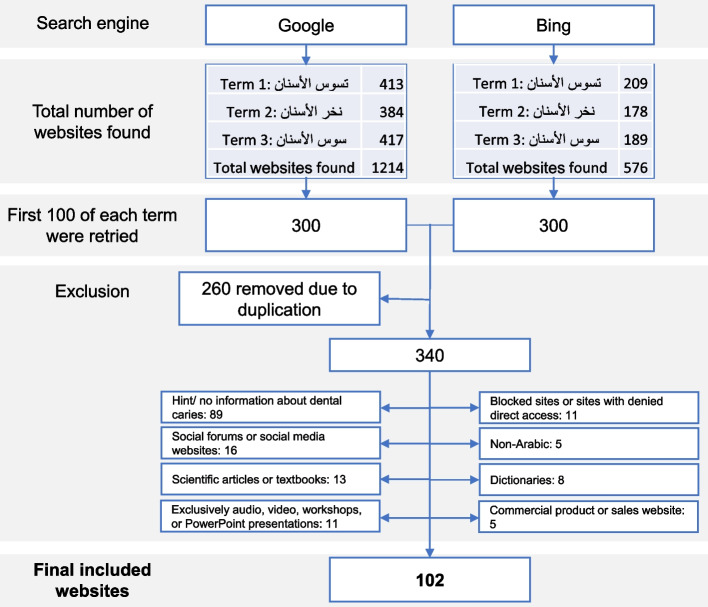
Flow chart of the process of finding websites using the search strategy

The three most common terms for dental caries that are equivalent in formal and slang Arabic were used, which are (تسوس الأسنان – نخر الأسنان – سوس الأسنان). The first 100 websites for each term were retrieved from both search engines, yielding 600 websites. All duplicate websites were removed, and the selection of resources for this study was subject to the following exclusion criteria: (1) social forums and social media websites; (2) complete scientific articles or textbooks; (3) exclusively audio, video-based resources, workshops, or PowerPoint presentations; (4) blocked sites or sites with denied direct access, requiring an ID and password; (5) non-Arabic-language sources; (6) dictionaries; (7) exclusive commercial product material found on sales websites like Amazon; and (8) sources with no or only minimal information about dental caries. These criteria were applied to ensure that only relevant and reliable information was included in the study. The selection process is shown in Fig. 1.

Each website was classified in terms of affiliation, specialization, and content type [37]. The affiliation had five distinct categories: commercial, nonprofit organization, university/medical center, government, and journalism. The specialization could be partial or exclusive to the topic. In terms of the content type, the website could contain medical facts, clinical trials, questions and answers, or human stories. Also, each website was recorded if it included video, audio, and/or images appropriate.

### Quality assessment

To evaluate the quality of websites, the assessment was carried out by two authors (referred to as AA and RA), both of whom are qualified dentists. The DISCERN and JAMA tools were employed in this process. Initially, calibration was completed in two stages. First, each author independently evaluated five websites, and any disparities in their assessments were resolved through discussion with the principal investigator. Second, 10 websites were evaluated and resolved, again, by discussion. Subsequently, inter-examiner calibration was computed for all the websites to ensure consistency in the evaluation process between the two examiners. For a disputed website, resolution was achieved with the principal investigator.

Each website was assessed using JAMA benchmarks [38], the presence of the HONcode [39], and the DISCERN tool [33]. JAMA benchmarks contained four main criteria to be fulfilled: (1) authorship (presence of author contributors, affiliations, and their relevant credentials), (2) attribution for references and citations, (3) disclosure (indications of sponsorship, ownership, commercial funding, advertising, and/or any likely conflicts of interest), and (4) currency (presence of date of publication and any updates). Each one of the previous criteria had a score of 1 if fulfilled, or 0 if not, for each website. The JAMA benchmark score was the sum of the previous items, ranging from 0 to 4 points (the highest score). The HONcode tool provides a mechanism by which websites can apply for permission to display the HONcode badge on the site, signifying that the site adheres to the HONcode criteria. This badge is valid for a duration of one year.

The DISCERN instrument is composed of a series of 16 inquiries that are categorized into three distinct sections. The initial segment, comprising questions 1 through 8, evaluates the reliability of websites as sources of information about specific therapies. The subsequent segment, comprising inquiries 9 through 15, pertains to diverse facets of therapeutic alternatives. Question 16 is an evaluative metric for comprehensive quality assessment. Each inquiry is allocated a numerical rating between 1 and 5, with a score of 1 denoting a website of substandard quality and a score of 5 signifying a website of superior quality. The DISCERN tool has a potential score range of 16 to 80. Websites that attain a score of 65 or higher are categorized as high quality, 33 to 64 are moderate quality, and websites that score in the range of 16 to 32 are deemed to be of low quality.

### Readability

Readability refers to the systematic application of formulae in order to determine the level of reading comprehension required to comprehend written text [40]. Because these websites should be understood by the general public, studies in the literature have indicated that a site should be easy to read and uncomplicated to a person who completed grade 6 in school [41]. The readability was assessed using a free readability calculator used primarily to assess English text https://www.online-utility.org/english/readability_test_and_improve.jsp. However, it has previously been used to assess dental websites in Arabic, as well [28–31]. The text of each website was copied and pasted into the readability calculator rather than having the calculator use a link to the site to retrieve the text. Only three indexes from this calculator were used: Flesch–Kincaid Grade Level (FKGL), Simple Measure of Gobbledygook (SMOG), and Flesch reading ease (FRE). The other indexes were not applicable to Arabic. The FKGL assesses the mean sentence length and mean syllables per word to ascertain a grade level of reading difficulty. The SMOG Index computes the proportion of words of three or more syllables; the higher the score, the greater the difficulty in reading. An acceptable readability score for the FKGL or SMOG is less than 7 [41, 42]. FRE calculates a score ranging from 0 to 100 based on the average length of sentences and the average number of syllables per word. An FRE score equal to or greater than 80 indicates acceptable readability [41, 42].

### Statistical analysis

Data entry and cleaning were completed with Microsoft Excel software 2023 v.2309 (Microsoft Corp, Redmond, WA). SPSS version 29 (IBM Corp., Armonk, NY) was used to analyze the data. Descriptive statistics were calculated for websites using counts, percentages, means, and standard deviation (SD). Spearman’s correlation was used to assess the correlation between JAMA, DISCERN scores, and readability indices, with a *p*-value of < 0.05 for significance. Kruskal Wallis and Mann–Whitney U were used for non-parametric tests.

## Results

### Number of websites dealing with dental caries

Using the search strategy detailed in Fig. 1 with the Arabic translation of “dental caries” generated 622, 562, and 606 websites for term1, term 2, and term 3, respectively, in the Google and Bing search engines, for a total of 1,790 websites. Out of the first 600 websites, using the three search terms and two search engines resulted in 102 websites remaining after excluding 260 duplicates; 89 websites with minimal or no information about dental caries; 13 social forums or social media websites; 16 scientific articles or textbooks links; 11 websites that were exclusively audio, video-based, a workshop, or a PowerPoint presentation; 11 blocked sites or sites with denied direct access; 5 non-Arabic websites; 8 dictionary websites; and 5 websites for commercial products or that were sales websites. The websites used in the analysis can be found in supplemental file S1.

The distribution of dental caries websites in terms of affiliation; specialization; content type; and sites consisting of an image, video, or audio content only is shown in Table 1.

**Table 1:** Description of websites based on affiliation, specialization, content type, presence of image, video and audio

**Variable**	**Category**	***n***	%
Affiliation	Commercial	57	55.88
	Journalism	23	22.55
	University/medical center	18	17.65
	Government	2	1.96
	Nonprofit organization	2	1.96
Specialization	Exclusively on topic	96	94.12
	Partly related to the topic	6	5.88
Content type	Medical facts	100	98.04
	Question and answers	2	1.96
	Clinical trials	0	0
	Human interest stories	0	0
Image	Yes	92	90.2
	No	10	9.8
Video	Yes	13	12.75
	No	89	87.25
Audio	Yes	1	0.98
	No	101	99.02

### Arabic dental caries websites quality assessment

When the JAMA benchmark criteria were checked, only 3.92% of the examined sites fulfilled the authorship criterion, 12.75% fulfilled attribution, 1.96% fulfilled disclosure, and 17.65% fulfilled currency, as shown in Table 2. A total of 67.65% of the websites did not fulfill any criteria at all, while 28.43% fulfilled 1 criterion, and 3.92% fulfilled 2 criteria. None of the websites scored 4 or 3 points on the JAMA benchmark criteria. The JAMA benchmark criteria had a mean score of 0.36 (SD = 0.56) and a median score of 0.

**Table 2:** Assessment of websites quality based on JAMA benchmark criteria

**JAMA benchmark criteria**	***n***	**%**
Fulfilled authorship	4	3.92
Fulfilled attribution	13	12.8
Fulfilled disclosure	2	1.96
Fulfilled currency	84	82.4
Met 0 criteria	69	67.7
Met 1 criteria	29	28.4
Met 2 criteria	4	3.92
Met 3 criteria	0	0
Met 4 criteria	0	0

None of the websites displayed the HONcode badge. With the DISCERN tool, the websites scored variably in each item, as shown in Table 3. According to DISCERN score classification, 33 (32.35%) websites had a low-quality score, 69 (67.65%) had a moderate-quality score, and none had a high-quality score. The highest scoring item was Q3 (relevance) and the lowest scoring item was Q8 (area of uncertainty).

**Table 3:** Arabic dental caries site scoring on DISCERN criteria

**Domain**	**Question**	**Mean**	**SD**
Reliability	Q1. Explicit aims	1.67	1.07
	Q2. Aims achieved	2.12	1.75
	Q3. Relevance	4.8	0.6
	Q4. Explicit sources	2.1	1.42
	Q5. Explicit date	2.5	0.99
	Q6. Balanced and unbiased	3.75	1.01
	Q7. Additional sources	2.69	1.73
	Q8. Areas of uncertainty	1.22	0.79
Treatment options	Q9. How treatment works	2.9	1.7
	Q10. Benefits of treatment	2.2	1.33
	Q11. Risks of treatment	1.33	0.85
	Q12. Effects of no treatment	2.98	1.92
	Q13. Effects on quality of life	1.02	0.2
	Q14. All alternatives described	3.27	1.95
	Q15. Shared decision	1.65	1.38
Overall rating	Q16. Overall rating	1.49	0.99
**Total DISCERN score**		**37.68**	**7.99**
Low score	*n* = 33 (32.35%)		
Moderate score	*n* = 69 (67.65)		
High score	*n* = 0 (0%)		

### Arabic dental caries websites readability

When the 102 Arabic dental caries websites were analyzed using readability calculators, the mean, SD, median, minimum, and maximum were recorded for each item, as shown in Table 4. A total of 52.94% had an FKGL of below 7, 91.18% had a SMOG below 7, and 85.29% had an FRE score of 80 or above.

**Table 4:** Readability measure for Arabic dental caries websites

**Variable**	***N*** (%)	**Mean**	**SD**	**Median**	**Min**	**Max**
Number of characters (without spaces):		6,972.70	14,535.80	3,536.00	886	103,565
Number of words		1,300.77	2,340.12	734.50	194	14,942
Number of sentences		89.01	258.51	27.00	3	1,730
Average number of characters per word		4.92	.44	4.84	4	8
Average number of syllables per word		1.04	.13	1.00	1	2
Average number of words per sentence		31.19	25.78	26.21	8	183
FKGL		8.84	9.91	6.70	0.67	68.14
FKGL < 7	54 (52.94)					
FKGL ≥ 7	48 (47.06)					
SMOG		4.03	1.68	3.00	3	10.23
SMOG < 7	93 (91.18)					
SMOG ≥ 7	9 (8.82)					
FRE		90.03	16.33	93.77	14.65	110.64
FRE < 80	15 (14.71)					
FRE ≥ 80	87 (85.29)					

*SD* Standard deviation, *FKGL* Flesch–Kincaid grade level, *SMOG* Simple Measure of Gobbledygook, *FRE* Flesch reading ease

Spearman’s correlation was calculated on the JAMA score, DISCERN score, number of words, number of sentences, FKGL, SMOG, and FRE (readability indexes), as shown in Table 5. The table shows a positive correlation between JAMA and DISCERN scores. There is also a positive correlation between DISCERN and the websites’ number of words and sentences. JAMA and DISCERN were not correlated with the readability indexes (FKGL, SMOG, or FRE).

**Table 5:** Spearman’s correlation of JAMA score, DISCERN score, and readability indexes

		DISCERN	Number of words	Number of sentences	FKGL	SMOG	FRE
JAMA	rho	0.344	0.113	− 0.01	0.173	0.048	− 0.154
	*p*-value	< .001	0.259	0.924	0.083	0.636	0.124
DISCERN	rho		0.37	0.286	0.039	0.171	− 0.075
	*p*-value		< .001	0.004	0.7	0.087	0.454
Number of words	rho			0.845	− 0.117	0.226	0.004
	*p*-value			< .001	0.242	0.023	0.965
Number of sentences	rho				− 0.571	0.084	0.463
	*p*-value				< .001	0.403	< .001
FKGL	rho					0.208	− 0.95
	*p*-value					0.037	< .001
SMOG	rho						− 0.35
	*p*-value						< .001

*FKGL* Flesch–Kincaid grade level, *SMOG* Simple Measure of Gobbledygook, *FRE* Flesch reading ease

Further analysis regarding the association between affiliation, specialization, content types, and the presence of images, videos, and audio with JAMA, DISCERN, number of words, number of sentences, FKGL, SMOG, and FRE can be found in supplemental file S2. The association was evaluated using the Kruskal–Wallis and Mann–Whitney U tests for non-parametric data.

## Discussion

Dental caries is one of the main oral health problems worldwide [2]. The internet is an important source of dental health information for the public [7–9], and to the best of our knowledge, infodemic studies about dental caries have not been previously conducted for Arabic or other languages. This study aimed to assess the quality and readability of websites related to dental caries in Arabic. The majority of the websites were commercial, exclusive to dental caries, and medical facts websites. The JAMA benchmark was low, and two-thirds of the sites did not fulfill any of the JAMA benchmark criteria. According to DISCERN, the majority of the sites were of moderate quality, but no websites were in the high-quality category. The readability indices were good, with half to a majority of websites scoring favorably to the cutoff points for FKGL, SMOG, and FRE. There was a positive correlation between JAMA and DISCERN scores, and DISCERN had a positive correlation to the websites’ number of words and sentences. However, neither JAMA nor DISCERN were correlated with the readability indexes (FKGL, SMOG, or FRE).

The mean of DISCERN in our study was similar to previous infodemic studies in Arabic that investigated periodontal diseases [28] and dental implants ([31], but higher than a study investigating denture hygiene [30]. The reason for the difference in the latter might be due to the last analysis investigating only 14 websites and, thus, more prone to error. Conversely, our JAMA total score was lower than all previous infodemic studies on periodontal disease topics in Arabic [29], denture hygiene [30], and dental implants [31]. The reason for this might be due to differences in the topic or potential differences in scoring JAMA criteria during the assessment. In fact, the JAMA criteria published in 1997 had relatively less clarity in scoring compared to the DISCERN scoring system, which contains many examples. Nevertheless, and despite the differences in scoring, the JAMA and DISCERN scores for Arabic websites on dental issues are unsatisfactory.

None of the websites had the HONcode badge. However, there was one website (https://www.mayoclinic.org/ar/diseases-conditions/cavities/symptoms-causes/syc-20352892), that initially had the code, but it was no longer displayed the next time the site was checked during the assessment. The previous website belonged to the Mayo Clinic, an American institution. This could have occurred because the time period for the HONcode badge had expired, but it is similar to previous infodemic studies investigating dental websites in Arabic, where each study found only one website [30, 31] or two [28] displaying the HONcode badge. However, this result is lower than similar dental infodemic studies in English, where the percentage ranges from 6.7% to 17% [17, 21, 43]. Nevertheless, as the HONcode seems to be directed more to English-language content and websites, the organization and verification might not be a point of concern among Arabic users or Arabic content providers.

Our study is in agreement with previous Arabic infodemic studies (investigated periodontal diseases, dental implants and denture hygiene) that the majority of the websites are readable [28, 30, 31], and the scores for FKGL, SMOG, and FRE are similar. This is in contrast to a previous study in English about oral manifestation of systematic sclerosis [20]. However, the ease of readability does not influence the quality of the websites as previously discussed. This study urges institutional organizations and universities to provide more reliable sources of information about dental caries, given that it is the most common oral disease and affects a large proportion of people around the world, and specifically, Arabic-speaking populations. This is important, because patients are reported to have many barriers when searching the internet, including the low potential of evaluating the written material [44, 45].

This study excluded social media sites, despite the fact that many people use social media as sources of information [46, 47] because it is much easier, interactive, and more enjoyable. Thus, future studies might assess the quality, readability, and reachability of Arabic content about dental caries on social media sites.

It should be noted that the quality assessment of this study did not focus on the content of these websites, but rather, focused on the existence of several factors that make the website more reliable as a source of information. One of the noteworthy aspects of this study is that many websites spread information about herbal home remedies for curing dental caries completely without the intervention of a dentist. It is crucial that further studies be conducted assessing the quality of the content of Arabic-language dental caries websites.

## Conclusions

The current Arabic websites discussing dental caries are low in quality, despite being generally easily readable. This urges the need to enhance the Arabic content related to dental caries for Arabic users. While the readability ensures that a broad range of Arabic users can access information about caries, the lack of credible information could result in misinformation or misconceptions about dental caries. More studies are needed to assess the content of oral health and disease sites in the Arabic language, as they are limited. Also, organized effort is needed to introduce more reliable sources discussing dental caries and treatment options to supply Arabic users with proper information.

### Supplementary Information

**Additional file 1: Supplementary file 1.** Raw data.

**Additional file 2: Supplementary file 2.** Additional analysis. **S2.** The association between affiliation, specialization, content types, the presence of image, video and audio with JAMA, DISCERN, number of words, number of sentences, KFGL, SMOG and FRE.

## Data Availability

The supplemental file for this paper S1 contains all the data produced or analyzed during this investigation.

## Abbreviations

FKGL: Flesch–Kincaid Grade Level
FRE: Flesch reading ease
HON: Health on the Net
SMOG: Simple Measure of Gobbledygook
