# Supplementary material for: Quality and readability of web-based information on dental caries in Arabic: an infodemiological study
Source: BMC Oral Health. 2023 Oct 25;23:797. doi: 10.1186/s12903-023-03547-1 (PMC10601140; doi:10.1186/s12903-023-03547-1)
Supplement: Supplementary file 2 — Additional file 2: Supplementary file 2. Additional analysis. S2. The association between affiliation, specialization, content types, the presence of image, video and audio with JAMA, DISCERN, number of words, number of sentences, KFGL, SMOG and FRE. [file 12903_2023_3547_MOESM2_ESM.docx]

Supplementary file 2: Additional analysis

S2: The association between affiliation, specialization, content types, the presence of image, video and audio with JAMA, DISCERN, number of words, number of sentences, KFGL, SMOG and FRE

|  |  |  | JAMA | DISCERN total | Number of words | Number of sentences | FKGL | SMOG | FRE |
| --- | --- | --- | --- | --- | --- | --- | --- | --- | --- |
|  |  | N | Mean Rank | Mean Rank | Mean Rank | Mean Rank | Mean Rank | Mean Rank | Mean Rank |
| Affiliation** | Commercial | 57 | 57.07* | 54.26 | 53.26* | 50.42* | 56.37* | 56.04 | 45.67* |
|  | Government or Non-profit organization | 4 | 63.63 | 52.75 | 51.00 | 60.88 | 29.25 | 53.75 | 70.25 |
|  | University/medical Centre | 18 | 37.72 | 52.39 | 64.42 | 68.50 | 34.44 | 38.50 | 70.94 |
|  | Journalism | 23 | 46.37 | 43.74 | 37.11 | 39.24 | 56.65 | 50.04 | 47.48 |
| Specialization | Exclusively | 96 | 52.53 | 53.3* | 51.31 | 51.05 | 52.26 | 52.51 | 50.77 |
|  | Partly related to the topic | 6 | 35.00 | 22.67 | 54.50 | 58.67 | 39.33 | 35.42 | 63.17 |
| Content type | Medical facts | 100 | 51.34 | 51.58 | 51.76 | 51.46 | 51.92 | 51.95 | 51.00 |
|  | Question and answers | 2 | 59.50 | 47.50 | 38.50 | 53.50 | 30.50 | 29.00 | 76.50 |
| Had image | Yes | 92 | 50.98 | 51.65 | 53.41 | 52.26 | 52.51 | 52.95 | 50.26 |
|  | No | 10 | 56.25 | 50.15 | 33.95* | 44.50 | 42.20 | 38.20 | 62.90 |
| Had video | Yes | 13 | 57.62 | 49.00 | 40.12 | 40.19 | 60.35 | 54.77 | 45.00 |
|  | No | 89 | 50.61 | 51.87 | 53.16 | 53.15 | 50.21 | 51.02 | 52.45 |
| Had audio | Yes | 1 | 35.00 | 69.50 | 50.00 | 78.50 | 10.00 | 61.00 | 94.00 |
|  | No | 101 | 51.66 | 51.32 | 51.51 | 51.23 | 51.91 | 51.41 | 51.08 |

FKGL = Flesch–Kincaid grade level; SMOG = Simple Measure of Gobbledygook; FRE = Flesch reading ease.

*p<0.05

**Using Kruskal Wallis test. Other variables were assessed by Mann–Whitney U test.
